# The feasibility and effectiveness of a novel online mental health literacy course in supporting university student mental health: a pilot study

**DOI:** 10.1186/s12888-022-04139-z

**Published:** 2022-07-30

**Authors:** N. King, B. Linden, S. Cunningham, D. Rivera, J. Rose, N. Wagner, J. Mulder, M. Adams, R. Baxter, A. Duffy

**Affiliations:** 1 Department of Public Health Sciences, Queen’s University, Kingston, Canada; 2 Health Services and Policy Research Institute, Queen’s University, Kingston, Canada; 3 Department of Biomedical & Molecular Sciences, Queen’s University, Kingston, Canada; 4 Department of Pharmacology and Toxicology, University of Toronto, Toronto, Canada; 5 Department of Psychiatry, Queen’s University, Kingston, Canada; 6 Office of Professional Development & Educational Scholarship, Queen’s University, Kingston, Canada; 7 Centre for Research Collections, University of Edinburgh Main Library, University of Edinburgh, Edinburgh, UK; 8 Department of Psychiatry, University of Oxford, Oxford, UK

**Keywords:** Mental health, Health literacy, Health behavior, Healthy lifestyle, Students, University

## Abstract

**Background:**

There is a need for effective universal approaches to promote and support university student mental health that are scalable and sustainable. In this pilot study we assess the feasibility and acceptability of a fully-digitalized, comprehensive mental health literacy course co-created with and tailored to the needs of undergraduate students. We also explore preliminary associations with mental health and positive behaviour change.

**Methods:**

An accredited online mental health literacy course was developed using state-of-the-art pedagogical principles and a reverse mentorship approach. The course was offered as an interdisciplinary undergraduate elective. Students completed an online survey before and after the 12-week course that collected demographic information and assessed mental health knowledge, emotional self-awareness, mental health, stigma, and health-related behaviors using validated measures. Dependent group t-tests were used to compare pre- and post-course levels of knowledge, mental health, sleep quality and substance use. Mental health outcomes of students who completed the course were compared to an age and sex-matched sample of students not enrolled in the course and who completed the same survey measures over the same academic year. Multivariable linear regression was used to examine the effect of course participation on outcomes at follow-up.

**Results:**

The course had good uptake and was positively reviewed by participants. Specifically, students found the course engaging, relevant, and applicable, and agreed they would recommend it to their peers. Among course participants there was improvement in mental health knowledge (*p* < 0.001) and emotional self-awareness (*p* = 0.02) at course completion. Compared to the matched comparison group, taking the course was associated with reduced alcohol (*β* = − 0.41, *p* = 0.01) and cannabis use (*β* = − 0.35, *p* = 0.03), and improved sleep quality (*β* = 1.56, *p* = 0.09) at the end of the term.

**Conclusions:**

Findings suggest that delivering mental health literacy as an online accredited course may be an acceptable and effective way of promoting university student mental health through improved knowledge, emotional self-awareness, and healthy lifestyle choices. As the course is expanded to larger and more diverse student cohorts we will be able to further examine the short and long-term effectiveness of the course in supporting student mental health and the underlying mechanisms.

**Supplementary Information:**

The online version contains supplementary material available at 10.1186/s12888-022-04139-z.

## Background

Over the past decade, prevalence estimates for common mental disorders have increased among post-secondary students in line with similar trends observed among the general population of Canadian youth [1–3]. In parallel, there has been a steady increase in the demand for campus mental health support and in the proportion of incoming undergraduate students reporting a lifetime history of mental disorders [4]. These trends have been observed at universities around the world [5–7]. Due to the COVID-19 pandemic necessitating social distancing and remote learning, the need for mental health support amongst students may increase further [8, 9].

University coincides with a critical stage in biopsychosocial and academic development and presents an opportunity to develop healthy behaviours and socioemotional coping resources that promote well-being [1]. Positive mental health is a prerequisite to successful completion of higher education, which is itself a major driver of healthy societal growth [10]. Therefore, there is a recognized need for effective approaches to promote and support university student mental health that are scalable and sustainable. Once such approach is to improve mental health literacy (MHL), defined as the understanding of how to obtain and maintain positive mental health, awareness of mental disorders and their treatments, and when and where to seek mental health support [11]. Improving MHL may be an effective way to promote good mental health and support timely help-seeking possibly through a reduction in stigma [12] and improved emotional self-awareness [13]. There is evidence that MHL is effective in improving knowledge about mental health and reducing stigma among secondary school students [14, 15]. However, there is limited evidence about the feasibility and effectiveness of MHL among university students, along with a lack of knowledge about underlying mechanisms [16]. Recent efforts, including courses focused on positive psychology [17, 18], mental health first aid training [19], and self-help resources supporting the transition to university [20, 21] have demonstrated positive preliminary results, with short-term improvement in reported mental health knowledge and well-being.

In this article we describe the initial offering of a more comprehensive MHL course developed in partnership with university students. The course covers all domains of MHL as described by Kutcher and colleagues [22] including health promotion, understanding mental illness and treatment, reducing stigma, and improving help-seeking. It was designed as a fully accredited (i.e., counts toward degree completion), interdisciplinary (without pre-requisite learning background) elective (not mandatory for degree). In this pilot study, we aimed to determine if this course is a feasible and acceptable way to deliver mental health literacy to university students. Further, we explored whether the course is helpful in terms of promoting mental health and positive behaviour change.

## Methods

### Course development

“*The Science of Well-being, Mental Health and Resiliency*” was developed using state-of-the-art online pedagogical and equity, diversity, and inclusivity principles. In partnership with a team of education developers, instructional designers, and graphic designers, the course was developed utilizing pedagogical principles such as Bloom’s taxonomy to define appropriate learning outcomes [23]; constructive alignment to ensure content, learning outcomes, and assessments were aligned [24]; and Mayer’s principles of multimedia learning to guide the design of interactive module components [25]. The development process included a dedicated equity, diversity, Indigeneity, inclusion, and accessibility (EDIIA) review by an Indigenous pedagogy expert at the institution; an accessibility review by the team to ensure the modules exceed Accessibility for Ontarians with Disabilities Act (AODA) standards; and a production test to ensure functionality and successful integration with learning management systems. The course is organized into six learning modules and incorporates evidence across disciplines. The aim of the course is to provide students from varied learning backgrounds with a comprehensive, evidence-based understanding of how to optimize their mental health and well-being, recognize emerging mental health concerns, and seek timely and appropriate help. The curriculum covers mental health and well-being as viewed through a multidisciplinary lens, the developing young adult brain, biopsychosocial contributors to mental health, protective and risk factors, as well as health promoting behaviours (i.e., substance use, sleep, and stress management). In each course module, self-reflection exercises and fictional student stories provide opportunities for knowledge consolidation and application. A key component of the course development process was the use of reverse mentorship [26, 27], wherein current or newly graduated students from diverse learning backgrounds were included as key members of the development team. These students provided insight to the content experts and recommended teaching and assessment strategies for online learning to advance the educational content and student satisfaction. Additional details about the course can be found here.

### Study design

The design was a parallel group longitudinal study, that included Bachelor of Health Sciences students who took the course and an age and gender matched comparison group drawn from the U-Flourish Student Well-Being and Academic Success survey study [28]. Students who enrolled in the course for the winter semester (January–April 2021) completed an abbreviated version of the U-Flourish survey during the first week (pre-course) and last week (post-course) of the 12-week term. The content of the full U-Flourish survey and procedure for obtaining informed consent is described in detail elsewhere [28, 29]. Briefly, undergraduate students are invited to complete surveys sent to their university email to assess well-being and mental health risk and protective factors using validated measures at the start and end of each academic year.

The pre/post MHL course survey completed by course participants included a measure of mental health knowledge and the same validated measures as the broader U-Flourish survey to collect demographic data and assess well-being, mental health, emotional self-awareness, stigma, and health-related behaviors (Supplement A). On the post-course survey students were also invited to share feedback about the course in the form of both open text and ranked responses. As incentive to participate, students in the course were offered an additional 2% toward their overall course grade for completing both surveys. Students in the course who did not wish to participate in research were offered the opportunity to complete alternative self-reflection assignments before and upon completion of the course of equal effort to earn the same additional 2% grade credit.

To compare outcomes of interest to the broader student population, a 2:1 age and gender matched comparison sample was randomly selected from the larger U-Flourish survey cohort; this group attended the same university and completed the same set of core measures during the same academic year but were not enrolled in the MHL course. Surveys were linked by student email and then de-identified for data analysis by research team members not involved in course development or grading. All students participating in this research read a letter of information and electronically provided consent prior to completing the survey. Ethics approval was obtained from the Queen’s University Health Sciences and Affiliated Teaching Hospitals Research Ethics Board (HSREB PSIY-692-20).

### Measures

The measures are described in detail elsewhere [29]. Briefly, symptoms of anxiety and depression were evaluated using the total symptom scores on the Generalized Anxiety Disorder Scale (GAD-7) (score range 0–21) [30] and Patient Health Questionnaire (PHQ-9) (score range 0–27) [31], respectively. The Short-Form Warwick-Edinburgh Mental Well-being Scale (WEMWBS-7) was used to measure well-being (score range 0–28, with higher scores indicating higher well-being) [32]. Sleep quality was evaluated using the Sleep Condition Indicator (SCI-8) (score range 0–32, with lower scores indicating lower sleep quality and more sleep problems) [33], while frequency of alcohol and cannabis use in the past month was evaluated on a 6-point scale ranging from 0 (Never) to 5 (Every day or nearly every day). Binge drinking was defined as drinking 5 or more alcoholic drinks on one occasion and was measured on a 5-point scale from 0 (Never) to 4 (Daily or almost daily). Stigma was assessed using the Barriers to Care Evaluation (BACE-3) stigma subscale (range 0–27, with higher scores indicating greater perceived stigma) [34].

Several items important to course evaluation were added to the abbreviated U-Flourish pre/post-course surveys, including the recognition and identification subscales of the Emotional Self-Awareness Scale (ESAS) [35], and a modified version of the Mental Health Literacy Scale (MHLS) [36] (see Supplement B). The modified MHLS included 15 multiple choice questions, with one point awarded for each correct answer and responses summed to derive a total score (range 0–15). Two additional questions assessed students’ knowledge of mental health resources on a 5-point scale ranging from 1 (strongly disagree) to 5 (strongly agree). On the post-course survey, students were asked to rate their level of agreement with four statements concerning the course on a scale from 1 (strongly disagree) to 7 (strongly agree) and were invited to share qualitative feedback about their experience. Questions included: “the course helped me be aware of my well-being and mental health”, “the course was engaging and held my interest”, “I will be able to apply what I learned to my well-being and mental health”, and “I would recommend this course to other students”.

### Analysis

Students’ acceptability of the course were assessed using the framework technique [37], where two research assistants blind to the quantitative analysis reviewed open-text responses and identified and compared common themes, with a third party available to break ties. Descriptive statistics were calculated for all quantitative variables. Chi-squared tests were used to compare pre/post course distributions in course participants, and dependent group t-tests were conducted to compare mean pre/post-course scores at the beginning and end of term for both course participants and the matched comparison group. Effect sizes were estimated using Cohen’s *d* [38]. Multivariable linear regression analysis was used to evaluate associations between course participation and outcomes at post-course, adjusting for baseline outcome status. Person-mean imputation was used if a single item was missing for a given scale. All statistical analyses were performed using SAS Version 9.4 (SAS Institute).

## Results

### Demographics

Demographic characteristics of the samples are displayed in Table 1. The average age of course participants was 23.4 (SD = 9.1) and the majority of course participants were female (91.1%). Course participants described their ethnicity as white (66.7%), South Asian (11.1%), East or Southeast Asian (8.9%), Middle Eastern (4.4%), Black (2.2%), Latino (2.2%), or multi-ethnic (4.4%), an ethnicity breakdown broadly representative of the Queen’s undergraduate population [39]. The comparison sample was slightly younger on average (22.1 years, *SD* = 7.6) and had minor differences in ethnicity distribution. At the baseline pre-course assessment students who took the course reported less frequent binge drinking (*p* < 0.001) and cannabis use (*p* = 0.08), but also lower sleep quality (*p* = 0.02) and greater symptoms of depression (*p* = 0.12) on average compared to the age and sex matched comparison group drawn from the general Queen’s student population who participated in the U-Flourish survey study and did not enrol in the course.

**Table 1:** Demographic characteristics of course participants and the matched comparison group

	Course Participants (*n* = 45)		Matched Comparison Group (*n* = 90)	
	*n*	(%)	*n*	(%)
Age group				
18	14	(31.1)	28	(31.1)
19	11	(24.4)	22	(24.4)
20	3	(6.7)	6	(6.7)
21+	17	(37.8)	34	(37.8)
Age, *Mean (SD)*	23.4	(9.1)	22.1	(7.6)
Gender				
Male	4	(8.9)	8	(8.9)
Female	41	(91.1)	82	(91.1)
Ethnicity				
White	30	(66.7)	65	(72.2)
Black	1	(2.2)	1	(1.1)
East/ Southeast Asian	4	(8.9)	12	(13.3)
Latino	1	(2.2)	2	(2.2)
Middle Eastern	2	(4.4)	0	(0.0)
South Asian	5	(11.1)	2	(2.2)
Multiple	2	(4.4)	8	(8.9)

### Uptake and acceptability

For this first offering of the course, enrolment was capped at 50 students; all spots were filled within the first 2 weeks of the registration period. Following the “add/drop” period, 45 students remained enrolled and completed the course. Students reviews of the course were very positive, with average ratings ranging from 5.9 (*SD* = 1.2) to 6.3 (*SD* = 1.1) out of a possible score of 7. Students agreed that the course was engaging and held their interest, and that they would recommend the course to others. Further, they felt the course content increased self-awareness and could be applied to support their mental health and well-being. Three themes were identified in the qualitative feedback: (i) overall interest and engagement in course content, (ii) a positive perception that the university was prioritizing student mental health by offering the course, and (iii) the course was helpful in supporting health promoting behaviours. See Supplement C for more details on course uptake and acceptability.

### Effectiveness at promoting mental health and positive behaviour change

The following sections present preliminary findings on the effectiveness of the course at improving student mental health and well-being, and promoting positive behaviour change. Due to the small and select sample that participated in this initial course offering there was limited power to study such effects.

#### Knowledge, self-awareness, and stigma

After completing the MHL course participants reported significantly improved levels of mental health knowledge (*p* < 0.001) and emotional self-awareness (*p* = 0.02) (Table 2). A notable, large improvement in mental health knowledge was observed (Cohen’s *d* = 0.96). Further, course participants indicated that they were more likely to feel confident about where to seek dependable mental health information after completing the course (*p* = 0.07) (Table 3). While no statistically significant change in confidence on how to access help was identified, the proportion of course participants who were already confident in this regard was high (i.e., 78% pre-course and 82% post-course). Similarly, perceived stigma related to accessing mental health support was low at baseline and did not change after taking the course (Table 2).

**Table 2:** Changes in lifestyle and health behaviours, mental health and well-being outcomes from baseline (pre-course) to follow-up (post-course) in course participants and the matched comparison group, and the impact of course participation on outcomes at follow-up

	Course Participants (***n*** = 45)						Matched Comparison Group (***n*** = 90)						Associations between Course Participation and Outcomes at Follow-up		
Outcomes	Baseline		Follow-up				Baseline		Follow-up						
**Lifestyle and Health Behaviors**	Mean	(SD)	Mean	(SD)	p*	d**	Mean	(SD)	Mean	(SD)	p*	d**	***beta	(SE)	*p*-value
Sleep Quality (0–32)	17.6	(6.5)	18.4	(6.1)	0.25	0.17	20.3	(6.5)	18.9	(7.3)	0.01	0.27	1.56	(0.91)	0.09
Binge Drinking (0–4)	0.42	(0.69)	0.31	(0.56)	0.10	0.25	1.11	(1.02)	1.01	(1.13)	0.20	0.14	−0.14	(0.12)	0.24
Alcohol Use (0–5)	1.67	(1.49)	1.22	(2.78)	<.001	0.26	2.02	(1.36)	1.87	(1.24)	0.18	0.14	−0.41	(0.16)	0.01
Cannabis Use (0–5)	0.42	(0.87)	0.42	(1.01)	0.99	0.00	0.80	(1.29)	1.11	(1.49)	0.003	0.32	−0.35	(0.16)	0.03
**Mental Health and Well-being**															
Depressive Symptoms (0–27)	8.3	(6.5)	8.8	(6.3)	0.52	0.10	6.6	(5.7)	8.8	(6.4)	<.001	0.51	−1.29	(0.63)	0.12
Anxiety Symptoms (0–21)	8.2	(5.3)	9.3	(5.9)	0.11	0.23	8.8	(6.4)	9.3	(5.5)	<.001	0.10	−0.90	(0.80)	0.26
Mental Well-being (0–28)	16.0	(5.2)	15.2	(5.6)	0.24	0.18	15.9	(5.3)	15.3	(5.0)	0.24	0.15	−0.22	(0.73)	0.76
Mental Health Knowledge (0–15)	10.0	(1.5)	11.6	(1.6)	<.001	0.97									
Emotional Self-Awareness (0–44)	21.9	(4.4)	22.6	(5.4)	0.02	0.16									
Perceived Stigma (0–27)	7.4	(6.3)	7.4	(7.2)	0.93	0.00									

(1) *p-value from dependent group t-test of difference in means, (2) **Cohen’s d effect size for dependent group difference in means, (3) ***beta estimate obtained from multivariable linear regression, adjusting for baseline outcome status, (4) Sleep quality was measured using the SCI-8, with higher scores indicating greater sleep quality; Binge drinking was evaluated on a frequency scale ranging from 0 (Never) to 4 (Daily or almost daily); Alcohol and cannabis use were evaluated on a frequency scale ranging from 0 (never) to 5 (every day or nearly every day); Depressive symptoms were measured using the PHQ-9 and anxiety symptoms were measured using the GAD-7, with higher scores indicating greater symptoms; Mental well-being was measured with the SF-WEMWBS-7, with higher scores indicating greater well-being; Emotional self-awareness was measured using the ESA-11, with higher scores indicating greater emotional self-awareness; Perceived stigma was measured using the stigma subscale of the BACE-3, with higher scores indicating a greater level of perceived stigma

**Table 3:** Changes in mental health literacy in course participants (*n* = 45)

	Pre-Course		Post-Course		
	***n***	(%)	***n***	(%)	p*
Confident I know where to seek dependable information about mental health, *n(%) Agree*	32	(71.1)	39	(86.7)	0.07
Strongly disagree	1	(2.2)	0	(0.0)	
Disagree	2	(4.4)	1	(2.2)	
Neutral	10	(22.2)	5	(11.1)	
Agree	21	(46.7)	26	(57.8)	
Strongly agree	11	(24.4)	13	(28.9)	
Confident I know how to access mental health support if needed, *n(%) Agree*	35	(77.8)	37	(82.2)	0.60
Strongly disagree	0	(0.0)	0	(0.0)	
Disagree	3	(6.7)	2	(4.4)	
Neutral	7	(15.6)	6	(13.3)	
Agree	23	(51.1)	25	(55.6)	
Strongly agree	12	(26.7)	12	(26.7)	

(1)* *p*-value for chi-square test comparing the proportion who ‘Agree’ or ‘Strongly Agree’ at baseline and follow-up

#### Health-promoting behaviours

**Fig. 1:**
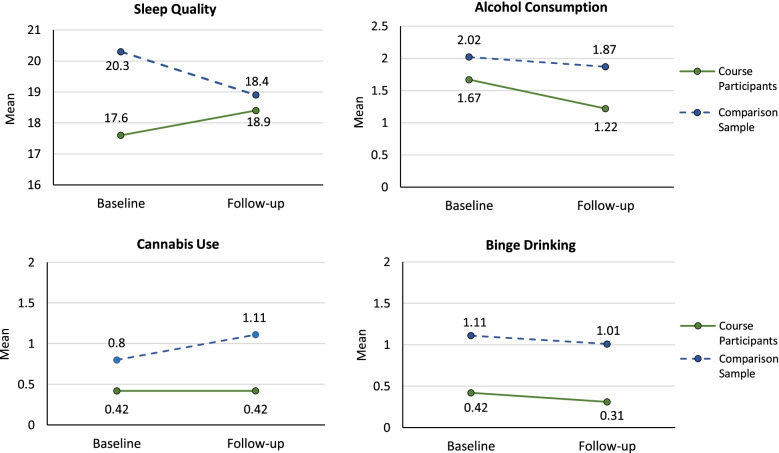
Changes in sleep quality (range = 0–32), past month alcohol and cannabis use (0–5), and binge drinking (0–4) from baseline (pre-course) to follow-up (post-course) in course participants (*n* = 45) and the matched comparison group (*n* = 90). Note that *p*-values are from a dependent group t-test of difference in means

Compared to the matched comparison group, participation in the MHL course was associated with improved sleep scores at the end of the term which fell short of statistical significance (*β* = 1.56, *p* = 0.09; Table 2). From the beginning to end of the term there was a non-significant increase in average sleep quality scores among course participants (*p* = 0.21), compared to a significant decrease in scores indicative of more sleep problems among the comparison student group (*p* = 0.01; Fig. 1). Further, course participants reported a significant reduction in alcohol (*β* = − 0.41, *p* = 0.01) and cannabis use (*β* = − 0.35, *p* = 0.03) at the end of term compared to the comparison group. Specifically, there was a significant decrease in alcohol consumption among course participants (*p* < 0.001) compared to a non-significant decrease among the matched comparison group (Table 2; Fig. 1). There was no change in the average frequency of cannabis use among course participants (*p* = 0.99), while an increase in average use was observed among the comparison student group (*p* = 0.003).

#### Mental health and overall well-being

Course participation was associated with a non-significant reduction in depressive symptoms at follow-up (*β* = − 1.29, *p* = 0.12). In students enrolled in the MHL course, average levels of depressive symptoms remained relatively high over time (*p* = 0.52) while there was a significant increase in average depressive symptom levels in the comparison group (*p* < .001). In both course participants and the comparison group there was a non-significant increase in average reported levels of anxiety symptoms and a non-significant decrease in average well-being scores from the beginning to end of the term. After adjusting for baseline symptom levels, course participation was not found to have a significant effect on self-reported symptoms of anxiety or well-being.

## Discussion

This pilot study provides preliminary evidence of good uptake and acceptability, and short-term improvement in knowledge, emotional self-awareness, and in health-promoting lifestyle behaviours observed in undergraduate students who completed this fully-digitalized MHL course co-created with students. These findings suggest that offering student mental health literacy as an online accredited course tailored for undergraduate university students may be an acceptable and effective way of promoting and supporting student mental health.

There is limited evidence about the effectiveness of teaching MHL to university students, as well as limited knowledge about the best method of delivery, what the important underlying mechanisms might be in supporting student mental health, and about the variation in engagement and benefit across diverse student groups. In response to these gaps and in partnership with students, we developed a comprehensive online MHL course tailored to undergraduate students. An optional research arm in conjunction with the U-Flourish Student Well-Being Survey Study will allow us to evaluate the impact of the course on indicators of student mental health and well-being, and potential mechanisms driving changes in these indicators. Preliminary results suggest this MHL course is an acceptable way to engage students. Course enrolment was rapid and student feedback positive in terms of the course being interesting and relevant, helpful in understanding the determinants of well-being and mental health and effective in supporting positive mental health and health-behaviours. Students learned how to identify the early warning signs of a mental health concern and were educated on appropriate help-seeking. We observed a significant increase in mental health knowledge and an improvement in emotional self-awareness in students who took the course. We also found preliminary evidence of an association between taking the MHL course and a reduction in substance use and sleep problems.

This study adds to the existing literature in that previous attempts to deliver MHL to post-secondary students have been limited to self-help resources, such as *Transitions,* a digital guide designed to provide incoming post-secondary students with a toolbox to support the transition to university [20] or one-off training sessions, such as *The Inquiring Mind: Post-Secondary,* a three-hour workshop intended to provide university students with practical knowledge about mental health [21]. Formal evaluations of these resources using small convenience samples did not show sustained long-term effects, and focused only on the limited outcomes of stigma, resiliency, and mental health knowledge [21, 22, 40]. Two curriculum-based efforts that we are aware of, *The Science of Well-being* [18], and the *Science of Happiness* [17], focus on psychoeducation and positive psychology. To our knowledge, no research has been conducted on the effects of the former curriculum-based approach. While an evaluation of the *Science of Happiness* demonstrated short-term improvements in overall well-being, sustained long-term effects were not observed, with scores regressing to baseline levels over time [17].

### Strengths and limitations

Findings from this study are preliminary and derive from the first offering of the course to a small sample of predominantly female students, enrolled in a single program, who self-selected into the course. As a result, these students may be highly invested in their mental health, and their experiences may not reflect those of the broader undergraduate student population. In addition, this MHL course was launched during the COVID-19 pandemic; though developed as an online course, the sustained impact of this global event on student mental health and higher education is largely unknown. Furthermore, students enrolled in the first offering of this course were relatively healthy at baseline given their reported sleep, substance use, stigma and symptom scores, producing a ceiling effect and limiting room for improvement in some outcomes of interest. Finally, given that this was a pilot study, we did not evaluate the observed effects over the longer term nor did we have sufficient power to test the hypothesized underlying mechanisms, which is a priority moving forward. We also had limited power to detect main effects, particularly in the course group; the decrease in average anxiety symptom scores was greatest in course participants based on the effect size estimate, but only statistically significant in the control group.

The course is currently being expanded and offered to students across different learning programs and universities both in Canada and in the UK. Subsequent offerings will therefore result in much larger and more heterogeneous student samples, which will be more representative of the student population across programs and institutions. The resulting student samples will provide sufficient variability in demographics and program of study, as well as baseline health-related behaviors, mental health knowledge, stigma, well-being and symptoms to examine the effects in diverse student populations and test mechanisms of action over the short- and longer-term.

That said, our MHL course had several strengths over previous interventions and course offerings to post-secondary students. State-of-the-art online pedagogical techniques and a reverse mentorship approach to capture the student perspective were used to develop the course. The course was also designed to be more comprehensive, covering all domains of mental health literacy as discussed by Kutcher et al. [22], and took an interdisciplinary approach, making it suitable for students across diverse learning backgrounds. Furthermore, the course evaluation included validated pre- and post-course measures of well-being, mental health outcomes, and putative mechanisms, which we will prioritize analyzing moving forward as our sample size allows. Finally, we were able to compare outcomes to a parallel matched comparison group. Other researchers [17] have used a staggered approach, where they assigned half of a group of students to take the course in the first semester, and the remainder to a “waitlist” condition (these students would take the course the following semester), but this has its own inherent biases as we know from previous research that mental health status changes over the course of a semester [41]. Our parallel comparison group, drawn from the same base population (i.e., undergraduate students who studied at the same university, over the same time period), provided us with the ability to look at differences between students who did and did not take the mental health literacy course. We were also able to match the comparison and intervention group on age and gender.

## Conclusions

The findings presented here provide preliminary evidence to suggest a comprehensive and tailored online MHL course may be an acceptable and effective way of imparting mental health knowledge, improving emotional self-awareness and promoting health-related behaviours in undergraduate university students, at least in the short-term. Therefore, providing MHL as an online accredited undergraduate course across learning programs, might be a useful and important direction for university student mental health promotion efforts. Moving forward it will be important for future research to show positive and meaningful effects and sustained benefit in larger heterogeneous student samples. In addition, it will be important to understand how best to adapt and implement this course in different institutions and across diverse student subgroups. Finally, it will also be helpful to understand the mechanisms underlying the observed effects on student outcomes and to work out what works best in terms of delivering mental health literacy, for whom, and why.

## Supplementary Information

**Additional file 1: Supplemental File A.** Table of Measures for MHL Course Survey. **Supplemental File B.** Modified Mental Health Literacy Scale (O’Connor et al., 2015) [36]. **Supplemental File C.** Course Uptake and Acceptability.

## Data Availability

The datasets analysed in this study are available from the corresponding author on reasonable request.

## Abbreviations

MHL: Mental health literacy
GAD-7: Generalized Anxiety Disorder Scale
PHQ-9: Patient Health Questionnaire
WEMWBS-7: Warwick-Edinburgh Mental Well-being Scale
ESAS: Emotional Self-Awareness Scale
MHLS: Mental Health Literacy Scale
SD: Standard deviation
