# Supplementary material for: The feasibility and effectiveness of a novel online mental health literacy course in supporting university student mental health: a pilot study
Source: BMC Psychiatry. 2022 Jul 30;22:515. doi: 10.1186/s12888-022-04139-z (PMC9338643; doi:10.1186/s12888-022-04139-z)
Supplement: Supplementary file 1 — Additional file 1: Supplemental File A. Table of Measures for MHL Course Survey. Supplemental File B. Modified Mental Health Literacy Scale (O’Connor et al., 2015) [36]. Supplemental File C. Course Uptake and Acceptability. [file 12888_2022_4139_MOESM1_ESM.docx]

**Supplemental File A. Table of Measures for MHL Course Survey**

| **Question Section** | **Baseline (Pre-Course)** | **Follow-up #1 (Post-Course)** | **Follow-up #2 (8 weeks Post-Course)** |
| --- | --- | --- | --- |
| *About You* | E-mail & Student # | E-mail & Student # | E-mail & Student # |
|  | Age | Age | Age |
|  | Gender | Gender | Gender |
|  | Ethnicity | Ethnicity | Ethnicity |
|  | Domestic student | Domestic student | Domestic student |
| *Emotional Health* | Ever dx with MI or learning problem | Ever dx with MI or learning problem | Ever dx with MI or learning problem |
|  | Age at time of dx | Age at time of dx | Age at time of dx |
|  | Lifetime tx for MI | Lifetime tx for MI | Lifetime tx for MI |
|  | Type of tx received | Type of tx received | Type of tx received |
|  | Lifetime ER visit for MI | Lifetime ER visit for MI | Lifetime ER visit for MI |
|  | Suicide screen | Suicide screen | Suicide screen |
| *Wellbeing and Mental Health Literacy* | Self-rated mental health | Self-rated mental health | Self-rated mental health |
|  | GAD-7 | GAD-7 | GAD-7 |
|  | PHQ-9 | PHQ-9 | PHQ-9 |
|  | WEMWBS-7 | WEMWBS-7 | WEMWBS-7 |
|  | PSS-4 | PSS-4 | PSS-4 |
|  | SCI-8 | SCI-8 | SCI-8 |
|  | Social Support/Competence Subscales | Social Support/Competence Subscales | Social Support/Competence Subscales |
|  | ESA-11 | ESA-11 | ESA-11 |
|  | SCS-SF | SCS-SF | SCS-SF |
|  | School Connectedness Subscale | School Connectedness Subscale | School Connectedness Subscale |
|  | Barriers to Care Checklist | Barriers to Care Checklist | Barriers to Care Checklist |
|  | Stigma Subscale | Stigma Subscale | Stigma Subscale |
|  | Mental Health Knowledge Questions | Mental Health Knowledge Questions | Mental Health Knowledge Questions |
|  | Know where to seek info about MH | Know where to seek info about MH | Know where to seek info about MH |
|  | Know how to access MH support | Know how to access MH support | Know how to access MH support |
| *Lifestyle and Habits* |  |  |  |
|  | Freq of 5+ alcoholic drinks | Freq of 5+ alcoholic drinks | Freq of 5+ alcoholic drinks |
|  | Freq of substance use (past month) | Freq of substance use (past month) | Freq of substance use (past month) |
|  | Freq of health behaviors (past month) | Freq of health behaviors (past month) | Freq of health behaviors (past month) |
|  | Freq of self-care | Freq of self-care | Freq of self-care |
|  | Freq of screen time (hrs per day) | Freq of screen time (hrs per day) | Freq of screen time (hrs per day) |

**Notes.** (1) MI = mental illness; MH = mental health; tx = treatment; dx = diagnosis; (2) GAD-7 – Generalized Anxiety Disorder 7-item; (3) PHQ-9 – Patient Health Questionnaire 9-item; (4) WEMWBS-7 – Warwick-Edinburgh Mental Wellbeing Scale 7-item; (5) PSS-4 – Perceived Stress Scale 4-item; (6) SCI-8 – Sleep Condition Indicator; (7) 5-item social support and social competence subscales from RSA – Resilience Scale for Adolescence; (8) ESA-11 – Emotional Self-Awareness Scale; (9) SCS-SF – Self-Compassion Scale Short Form 12-items; (10) 4-item school connectedness subscale from CSSWQ - College Student Subjective Well-being Questionnaire; (11) 9-item stigma subscale from BACE-3 - Barriers to Care Evaluation.

**Supplemental File B. Modified Mental Health Literacy Scale (O’Connor et al., 2015) [36]**

Correct answers are **highlighted and bolded** here and are scored as ‘1’ while all other responses are scored as ‘0’. Sum all together for total score.

**The purpose of the following questions is to gain an understanding of your knowledge of various aspects related to mental health.**

| 1. **Which of the following is true about mental health problems in university students:**   **◯ More students are requesting learning accommodations for mental health reasons**  ◯ Suicide and self-harm rates in students are higher than in the general population of young people the same age  ◯ Most students require both medication and psychological therapy to improve  ◯ Rates of mental illness in students are higher than in the general population of young people the same age  ◯ Only a and c above   1. **Which of the following is true about the effects of stress?** |
| --- |
| ◯ Stress can result in anxiety and impaired performance  ◯ Stress can help individuals to perform optimally  ◯ Stress can result in physical manifestations, including fatigue and trouble sleeping  **◯ All of the above**  ◯ Only a and c above   1. **Which of the following is an example of self-care?**   ◯ Taking a walk  ◯ Experiencing or creating art  ◯ Taking some time to relax at the end of your day  **◯ All of the above**  ◯ Only a and c above   1. **It is possible to have a diagnosed mental illness and still experience good well-being.**   **◯ True**  ◯ False   1. **If someone is experiencing poor mental health, then they have a mental illness.**   ◯ True  **◯ False**   1. **What proportion of Canadian post-secondary students do you think report having been diagnosed or treated by a professional for one or more mental health condition(s)?**   ◯ 10%  **◯ 25%**  ◯ 35%  ◯ 45%  ◯ Over 50%   1. **What proportion of Canadian post-secondary students do you think report experiencing symptoms of anxiety?**   ◯ 25%  ◯ 45%  ◯ 55%  **◯ 65%**  ◯ 75%   1. **What proportion of Canadian post-secondary students do you think report experiencing symptoms of depression?**   ◯ Less than 20%  ◯ 20%  **◯ 45%**  ◯ 65%  ◯ 75%   1. **What proportion of Canadian post-secondary students do you think report binge drinking (5+ drinks at a single social event)?**   ◯ less than 10%  ◯ 20-25%  **◯ 30-40%**  ◯ 40%  ◯ Over 50%   1. **Which of the following has been associated with binge drinking in Canadian post-secondary students?**   ◯ accidents and injuries  ◯ unprotected sex and regretful decisions  ◯ blacking out and memory loss  ◯ worsened anxiety and depressive symptoms  **◯ All of the above** |

1. **Which of the following can be a long-term effect of excessive alcohol use?**

◯ Heart arrhythmias

◯ Disease of the liver, pancreas, and/or stomach

◯ Alcoholic dementia

**◯ All of the above**

◯ Only a and c

1. **According to the MacLeans 2019 Canadian University Survey on Marijuana Use: Queen’s University, what percentage of students reported using cannabis at least once per month?**

◯ 10%

**◯ 24%**

◯ 31%

◯ 48%

◯ over 50%

| 1. **Which of the following is associated with regular cannabis use?** |
| --- |

◯ Low motivation and cognitive dysfunction (i.e. memory loss, slowed thinking)

◯ Increased risk of psychotic symptoms (hallucinations, paranoia)

◯ Physical health problems including of the heart and lungs

**◯ All of the above**

◯ Only a and c

1. **Which of the following increases the risk of poor mental health and well-being in post-secondary students:**

◯ Taking time out of studies to exercise or enjoy hobbies

◯ Pushing yourself out of your comfort zone and trying new things

◯ Spending time in nature

◯ Creative screen time

**◯ None of the above**

1. **What does “mindfulness” mean?**

◯ Mindfulness is the same as meditation

**◯ Mindfulness improves self-awareness without judgement**

◯ You have to be good at sitting still to effectively practice mindfulness.

◯ There are no long-term benefits to practicing mindfulness.

◯ Mindfulness is about switching off your brain

**Supplemental File C. Course Uptake and Acceptability**

For the first offering of the course, enrolment was capped at 50 students; all spots were filled within the first two weeks of registration. Following the “add/drop” period, 45 students remained enrolled and completed the course Students reviews of the course were very positive, with average ratings ranging from 5.9 (*SD*=1.2) to 6.3 (*SD*=1.1) out of a possible score of 7 (Table 1).

| ***Table 1. Student Feedback on Course Content*** | | |
| --- | --- | --- |
| **Question** | **Mean** | **(SD)** |
| This course helped me to be more aware of my wellbeing and mental health. | 6.0 | (1.1) |
| This course was engaging and effectively held my interest. | 5.9 | (1.2) |
| I will be able to apply what I learned from this course to my wellbeing and mental health. | 6.1 | (1.1) |
| I would recommend this course to other students. | 6.3 | (1.1) |

Note: Students rated their level of agreement with each of these statements on an adjectival scale ranging from 1 to 7.

Students were also invited to share qualitative feedback about their experience with the course. Three themes were identified: (i) overall interest and engagement in course content (*n*=9), (ii) a positive perception that the university was prioritizing student mental health (*n*=3), and (iii) the course was helpful in supporting healthy behaviours (*n*=9). Demonstrative quotes are displayed below (Table 2).

***Table 2. Extracted Quotations***

| **Participant** | **Quote** |
| --- | --- |
| 001 | I really enjoyed that this course was evidence-based, all the information seemed credible and accurate, making me more likely to apply what I learned to my life. I liked the range of topics that were covered, and that it was strongly related to life as a university student, yet easily applicable beyond university. I also found that it gave me more hope, because I now feel like there's people out there acknowledging that the transition to university is tough and there are aspects of the mental healthcare system that need to be changed in order to better support students. I felt like the course was put together with compassion and care, making me feel more comfortable that I have more tools to improve my mental health and wellbeing, and that I know where to seek help if I need to. |
| 002 | I really enjoyed it and found that it was very helpful and enlightening. Thank you! |
| 003 | In this course, I have learned a lot about what it takes to be content in my own mind, and I can take these strategies with [me] throughout my life. |
| 004 | This was an amazing course and definitely helped educate enrolled students about mental health literacy. |
| 005 | I enjoyed learning about mental health and resilience especially among university students! |
| 006 | I loved taking this course!! I have recommended it to my friends, at least one of whom already signed up for the summer! This unique, practical, and relevant course talked about issues that affect everyone and yet are not covered in any other course here at Queen's. This course has provided me knowledge about mental health and advice that I have actively implemented into my life. Overall, I am less stressed, accomplishing more, happier overall compared to when I started this course. |
| 007 | This course was amazing, It positively changed my perspective on mental health and wellbeing. |
